# Investigation of Microbial Diversity in Geothermal Hot Springs in Unkeshwar, India, Based on 16S rRNA Amplicon Metagenome Sequencing

**DOI:** 10.1128/genomeA.01766-15

**Published:** 2016-02-25

**Authors:** Gajanan T. Mehetre, Aditi Paranjpe, Syed G. Dastager, Mahesh S. Dharne

**Affiliations:** a National Collection of Industrial Microorganisms (NCIM), CSIR-National Chemical Laboratory (NCL), Pune, India; b Academy of Scientific and Innovative Research (AcSIR), New Delhi, India

## Abstract

Microbial diversity in geothermal waters of the Unkeshwar hot springs in Maharashtra, India, was studied using 16S rRNA amplicon metagenomic sequencing. Taxonomic analysis revealed the presence of *Bacteroidetes*, *Proteobacteria*, *Cyanobacteria*, *Actinobacteria*, *Archeae*, and OD1 phyla. Metabolic function prediction analysis indicated a battery of biological information systems indicating rich and novel microbial diversity, with potential biotechnological applications in this niche.

## GENOME ANNOUNCEMENT

Deccan basaltic geothermal hot springs are rich in sulfur and yet are unexploited for microbial ecology (1). The geographical location of Unkeshwar is latitude 19°34′ to 19°40′N and 78°22′ to 78°34′E longitude, with water temperatures ranging from 42°C to 60°C, located in Maharashtra, India.

Replicate water samples were collected during December 2012 in sterile containers, filtered through 0.22-µm-pore-size filters (Merck Millipore, India), and DNA extraction was performed using the RNA PowerSoil total RNA isolation kit (Mo Bio Laboratories, Inc., Carlsbad, CA, USA), according to the manufacturer’s protocol. DNA was enriched by the Multiple Annealing and Loop-Based Amplification Cycles (MALBAC) protocol (2) and then amplified by using primers spanning the V3 to V4 region of the 16S rRNA gene (3).

Paired-end sequencing of the library was performed on an Illumina MiSeq platform using 2 × 251-bp chemistry. The quality parameters for the obtained sequences were checked using the FASTQ quality filter (Phred quality [Q] <20). The resulting good-quality sequences were then overlapped into single longer reads using SeqPrep QIIME (4). Chimeras were removed using the program UCHIME, and all nonchimeric sequences were taken for picking operational taxonomic units (OTUs) using the program Uclust, with a threshold of 97% similarity (5, 6). A representative sequence was identified for each OTU and aligned against a Greengenes core set of sequences using the PyNAST program (7, 8) Taxonomic classification was performed using the RDP Classifier (9) and Greengenes (7) OTU databases. Further, alpha diversity was determined by calculating Shannon, Chao1, and observed species metrics (4). The rarefaction curve was generated for each of the metrics, and metric calculations were performed using the QIIME software (8).

A total of 1,360,637 raw reads were obtained, and 873,631 reads were considered for analysis (after filtering), from which a total of 6,684 OTUs were detected. They were checked for singleton OTUs (i.e., OTU has single reads), and 4,935 singletons were identified and removed. A total of 1,749 OTUs were used for taxonomy classification showing the dominant phyla of *Bacteroidetes*, *Proteobacteria*, *Cyanobacteria*, and *Actinobacteria*. Around 80% of the reads were assigned to *Bacteroidetes* and the other 20% to all other phyla. Two of the reads were also assigned to *Archaea* belonging to the *Cenarchaeum* genus (phylum *Thaumarchaeota*). Interestingly, some of the reads mapped to the OD1 phylum, which is known for small genome size (0.7 to 1.2Mbp) and large inventories of novel proteins (10). The rest of the OTUs were mapped to other and unknown phyla. The microbial diversity within the samples was also calculated by Shannon, Chao1, and observed species metrics used to measure the estimated observed OTU abundances, accounting for both richness and evenness.

To determine the accuracy of functional predictions using a Phylogenetic Investigation of Communities by Reconstruction of Unobserved States (PICRUSt), inferences were compared (11) to understand survival strategies and adaptation in extreme niches. The results revealed a wider range of genetic diversity involved in various essential processes, like genetic (translation, transcription, and repair) and environmental information signaling and processing, cellular processes (cell growth and death, cell communication, cell motility, transport, and catabolism), signal transduction and metabolism (of carbohydrates, amino acids, lipids, terpenoids, polyketides, cofactors, vitamins, xenobiotics, energy, and proteins and biosynthesis of secondary metabolites) and organismal systems.

### Nucleotide sequence accession number.

The sequence reads obtained in this study were deposited in the Sequence Read Archive (SRA) accession no. SRX1499015.
